# Supplementary material for: Cause of Death, Mortality and Occult Blood in Colorectal Cancer Screening
Source: Cancers (Basel). 2022 Jan 4;14(1):246. doi: 10.3390/cancers14010246 (PMC8750981; doi:10.3390/cancers14010246)
Supplement: Supplementary file 1 [file cancers-14-00246-s001.zip › cancers-1544732-supplementary.pdf]

Supplementary

Supplementary tables

Table S1. Causes of death and the corresponding icd-8 and icd-10 classification codes.

Cause of death	ICD-8 classification	ICD-10 classification
Colorectal cancer	153-154	C18-C20
Non-colorectal cancer	140-152, 155-209	C00-C17, C21-C99
Cardiovascular disease	390-458	I00-I99
Respiratory disease	460-519	J00-J99
Digestive disease	520-577	K00-K99
Endocrine-and hematological disease	280-289, 240-258	D00-D99, E00-E99
Neuropsychological disease	290-358	G00-G99
External conditions	800-999	S00-S99, T00-T99, U00-U99, V00-V99, W00-W99, X00-X99, Y00-Y99, Z00-Z99

Abbreviations: ICD, International Classification of Disease.

Table S2. Diseases and indications potentially contributing to digestive bleeding .

Diseases And Indications	ICD-8 classification	ICD-10 classification
Hemorrhage of anus and rectum	-	K625-K626
Inflammatory Bowel Disease	561, 563	K50-K52
Diverticular disease	562	K57
Hemorrhoids	455	I84
Colorectal fissures	565	K60
Gastrointestinal ulcers	531–534	K25–K28
Gastritis	535	K29

Abbreviations: ICD, International Classification of Disease.

Table S3. Cause of death, allowing for missing on education and income.

	Positive gFOBT (*n* = 1,389)	Negative gFOBT (*n* = 14,153)	HR (95% CI)	*P*-value	aHR (95% CI)*	*P*-value
**All-cause mortality**	1389 (100.00)	14,153 (100.00)	1.46 (1.37–1.56)	<0.001	1.25 (1.17–1.34)	0.000
**All-cause excl. CRC**	1286 (92.58)	13,722 (96.95)	1.35 (1.26–1.45)	<0.001	1.16 (1.09–1.25)	0.000
**Colorectal cancer**	103 (7.42)	431 (3.05)	5.21 (4.10–6.63)	<0.001	4.48 (3.76–6.08)	<0.001
**Non-colorectal cancer**	353 (25.41)	3729 (26.35)	1.41 (1.24–1.61)	<0.001	1.24 (1.09–1.42)	0.001
**Cardiovascular disease**	567 (40.82)	5948 (42.03)	1.48 (1.34–1.64)	<0.001	1.24 (1.12–1.37)	0.000
**Respiratory disease**	323 (23.25)	3264 (23.06)	1.34 (1.16–1.54)	<0.001	1.13 (0.98–1.30)	0.091
**Digestive disease**	78 (5.62)	695 (4.91)	1.15 (1.56–2.04)	0.003	1.33 (1.00–1.78)	0.048
**Endocrine-and hematological disease**	110 (7.20)	981 (6.93)	1.64 (1.30–2.07)	0.000	1.42 (1.12–1.79)	0.003
**External conditions**	46 (3.31)	548 (3.87)	1.16 (0.80–1.70)	0.435	1.01 (0.69–1.48)	0.964

*__Adjusted for__: Age, gender, income, education, bleeding at baseline, comorbidity at baseline. **Abbreviations**: gFOBT, guaiac fecal occult blood test; HR, Hazard Ration; aHR, adjusted Hazard Ratio NB. Each participant may occur with more than one cause of death in this table.
